# Safety and Immunogenicity of BNT162b2 in Antiretroviral Therapy–Treated People With HIV

**DOI:** 10.1093/ofid/ofag310

**Published:** 2026-06-21

**Authors:** Essack Mitha, Mezgebe Berhe, Judith Aberg, Christopher J Lucasti, Fadi Chalhoub, Nicholas Kitchin, Isabel Vogler, Stephen P Lockhart, Sina Fuchs, Jasmin Quandt, William C Gruber, Chris Webber, Marie-Cristine Kühnle, Alexander Muik, Bonny Gaby Lui, Maria J G T Vehreschild, Christoph Stephan, Meghan Bushway, Xia Xu, Todd J Belanger, James Trammel Price, Stefan Liebscher, Ruth Bailey, Susan Mather, Elizabeth M Adams, Svetlana Shpyro, Eleni Lagkadinou, Özlem Türeci, Kena A Swanson, Uğur Şahin, Alejandra Gurtman

**Affiliations:** Newtown Clinical Research, Johannesburg, South Africa; North Texas Infectious Diseases Consultants, Dallas, Texas, USA; Division of Infectious Diseases, Department of Medicine, Icahn School of Medicine at Mount Sinai, NewYork, New York, USA; South Jersey Infectious Disease, Somers Point, New Jersey, USA; Clinical Neuroscience Solutions, Jacksonville, Florida, USA; Vaccine Research and Development, Pfizer Ltd, Marlow, United Kingdom; Cellular Biomarker and Immunology Research, BioNTech SE, Mainz, Germany; Vaccine Research and Development, Pfizer Ltd, Hurley, United Kingdom; Cellular Biomarker and Immunology Research, BioNTech SE, Mainz, Germany; Cellular Biomarker and Immunology Research, BioNTech SE, Mainz, Germany; Vaccine Research and Development, Pfizer Inc, Pearl River, NewYork, USA; Vaccine Research and Development, Pfizer Ltd, Marlow, United Kingdom; Analytical Services, BioNTech SE, Mainz, Germany; ADC-IO & COVID Vaccine Innovation, BioNTech SE, Mainz, Germany; Infectious Disease Vaccines, BioNTech SE, Mainz, Germany; Department II of Internal Medicine, Infectious Diseases, Goethe University Frankfurt, University Hospital Frankfurt, Frankfurt am Main, Germany; Department II of Internal Medicine, Infectious Diseases, Goethe University Frankfurt, University Hospital Frankfurt, Frankfurt am Main, Germany; Translational Immunology, BioNTech US, Cambridge, Massachusetts, USA; Vaccine Research and Development, Pfizer, Collegeville, Pennsylvania, USA; Vaccine Research and Development, Pfizer Inc, Pearl River, NewYork, USA; Vaccine Research and Development, Pfizer, Collegeville, Pennsylvania, USA; Cellular Biomarker and Immunology Research, BioNTech SE, Mainz, Germany; Vaccine Research and Development, Pfizer Ltd, Hurley, United Kingdom; Worldwide Medical and Safety, Pfizer Inc, Collegeville, Pennsylvania, USA; Clinical Development, BioNTech US, Cambridge, Massachusetts, USA; Clinical Development, BioNTech US, Cambridge, Massachusetts, USA; Clinical Development, BioNTech US, Cambridge, Massachusetts, USA; Global Development Organization, BioNTech SE, Mainz, Germany; Helmholtz Institute for Translational Oncology Mainz (HI-TRON), German Cancer Research Center, Mainz, Germany; Vaccine Research and Development, Pfizer Inc, Pearl River, NewYork, USA; Global Development Organization, BioNTech SE, Mainz, Germany; Helmholtz Institute for Translational Oncology Mainz (HI-TRON), German Cancer Research Center, Mainz, Germany; Vaccine Research and Development, Pfizer Inc, Pearl River, NewYork, USA

**Keywords:** BNT162b2, COVID-19, PWH, SARS-CoV-2, vaccination

## Abstract

**Background:**

People with human immunodeficiency virus (HIV) are at increased risk of severe COVID-19 and are recommended for priority vaccination. We investigated the safety and immunogenicity of a 2-dose regimen of the mRNA vaccine BNT162b2 in people with HIV (PWH) on antiretroviral therapy (ART).

**Methods:**

Safety, reactogenicity, and immunogenicity data were obtained from PWH on ART ≥16 years enrolled in the phase 1/2/3 BNT162-02 trial/C4591001 (NCT04368728) between September and November 2020, which randomized participants (1:1) to receive BNT162b2 or placebo. Immunogenicity was also investigated in a substudy of the open-label phase 1/2 BNT162-01 trial (NCT04380701) in PWH aged 18–85 years and age- and sex- matched HIV-negative controls enrolled February to March 2021.

**Results:**

In total, 201 PWH were included from the BNT162-02 trial. Higher rates of local and systemic reactions were reported during the 7 days following both doses by participants administered BNT162b2 versus participants administered placebo, most notably pain at injection site, fatigue, headache, and chills. Most reactions were mild to moderate in severity. No serious adverse events occurred up to 1 month post–dose 2. Spike protein–binding and severe acute respiratory syndrome coronavirus 2 (SARS-CoV-2) neutralizing antibody responses were elicited in participants who received BNT162b2 and remained above prevaccination baseline levels through 6 months post–dose 2. In the BNT162-01 trial, all PWH (N = 15) seroconverted, with BNT162b2-induced SARS-CoV-2 neutralization titers and longitudinal T-cell responses similar to those observed in the HIV-negative cohort.

**Conclusions:**

Primary 2-dose vaccination with BNT162b2 in PWH was well tolerated and immunogenic with sustained responses up to 6 months, supporting vaccination in this priority population.

## Teaser

Data from the substudies of the BNT162-01 and BNT162-02 trials demonstrate that a 2-dose vaccination series with BNT162b2 has a tolerable safety profile and is immunogenic in people with HIV on antiretroviral therapy, thus supporting its use in this population.

Evidence suggests that people with human immunodeficiency virus (HIV) are at increased risk of severe outcomes, hospital admission, and death associated with coronavirus disease 2019 (COVID-19) compared with HIV-negative individuals, with outcome driven by the same risk factors identified for the general population, including sex, age and the presence of comorbidities [1, 2]. In general, vaccination programs are recommended for people with HIV (PWH); however, vaccine-induced immune responses vary in duration and may be suboptimal in this population [3]. Therefore, some vaccines, including those targeting severe acute respiratory syndrome coronavirus 2 (SARS-CoV-2), the causative virus of COVID-19, have specific recommendations related to HIV status. In particular, PWH with a CD4 cell count of <200 cells/mm^3^, with evidence of an opportunistic infection, not on HIV treatment, and/or with a detectable viral load require special considerations for COVID-19 vaccination. For immunocompromised adults, the current Centers for Disease Control and Prevention guidelines recommend a complete initial series with additional doses based on shared clinical decision-making [4].

BNT162b2 (Comirnaty), a lipid nanoparticle–formulated mRNA vaccine [5], demonstrated 95% protection against COVID-19 in individuals aged 16 years or older in the global phase 1/2/3 BNT162-02/C4591001 clinical trial (NCT04368728) [6] and received emergency use authorization in December 2020 [7]. Observational studies of virologically suppressed ≥18-year-old PWH who received a primary 2-dose series of BNT162b2 showed similar seroconversion rates (≥98%) [8–13] and humoral and cellular responses (measured via CD4^+^/CD8^+^ enzyme-linked immunosorbent spot [ELISpot] assay) [14] as HIV-negative controls, including comparable levels of neutralizing antibodies against pseudoviruses of the Alpha (B.1.1.7), Beta (B.1.351), and Gamma (P.1) variants of concern (VOCs), which were circulating at the time of the study [14]. Small-scale studies indicate a safety profile in PWH broadly comparable with that reported in pivotal trials of BNT162b2 vaccination [6, 8, 9, 11, 15]. To further elucidate the benefit-risk of COVID-19 vaccination in PWH, safety (including reactogenicity) and immunogenicity of BNT162b2 vaccination were investigated in PWH on antiretroviral therapy (ART) enrolled in the BNT162-02 (NCT04368728) and BNT162-01 (NCT04380701) trials.

## METHODS

### Study Design and Participants

#### BNT162-02

BNT162-02/C4591001 was a randomized, observer-blind, international, multicenter, placebo-controlled phase 1/2/3 trial that evaluated BNT162b2. In phase 3 of the study, participants aged ≥16 years were randomized 1:1 to receive 30 µg BNT162b2 as a primary 2-dose series or placebo, given approximately 21 days (d) apart (see [6] for study details). An amendment to the BNT162-02 trial protocol added phase 3 eligibility criteria for PWH and an exploratory objective to describe safety, immunogenicity, and efficacy in participants with stable HIV disease (defined as <50 copies/mL, CD4^+^ T-cell count >200 cells/mm^3^, and stable ART ≥6 months), who were not included in the primary analysis [6]. Blood samples were taken for immunogenicity testing prior to dosing on day 0 (pre–dose 1 [pre-D1]), 1 month post–dose 2 (D2 + 1m), and 6 months post–dose 2 (D2 + 6m).

#### BNT162-01

BNT162-01 was a phase 1/2 clinical trial that investigated the safety and immunogenicity of 4 prophylactic SARS-CoV-2 RNA vaccines, including BNT162b2, in healthy and immunocompromised adults (see [15] for enrollment criteria). A population expansion cohort was introduced within BNT162-01 by a protocol amendment to evaluate safety and long-term immune responses in immunocompromised adults aged 18–85 years, who received two 30-µg BNT162b2 doses 21 days (d) apart. Inclusion criteria for the PWH subcohort were a CD4^+^ T-cell count of ≥200 cells/L, body mass index (BMI) >19 and <30 kg/m^2^, and body weight of ≥50 kg at screening. Based on eligibility criteria and clinical experience, PWH were presumed to be mildly immunocompromised but otherwise healthy in the view of the investigator. Individuals with additional known risk factors for COVID-19 including the following, were not eligible: evidence of opportunistic infections; malignant complications; other organ manifestations consistent with advanced AIDS; or other conditions that would be considered a contraindication for vaccination. Blood samples were taken for immunogenicity testing prior to dosing on day 0 (pre-D1) and on days 7 (D1 + 7d), 21 (D1 + 21d, pre–dose 2), 28 (D2 + 7d), 35 (D2 + 14d), 42 (D2 + 21d), 49 (D2 + 28d, end of treatment visit), and 183 (D2 + 162d).µ

### Participant Consent

Both trials were performed in accordance with the principles of the Declaration of Helsinki and Good Clinical Practice and all participants provided written informed consent; the trials were approved by the appropriate regulatory authorities, institutional review boards, and ethics committees.

### Safety

Solicited local and systemic reactogenicity events occurring within 7 days of each vaccination were collected and graded according to the US Food and Drug Administration Center for Biologics Evaluation and Research guidelines on toxicity grading scales for healthy adult volunteers [16]. All adverse events (AEs) were collected through 1 month post–dose 2 and serious AEs (SAEs) through 6 months post–dose 2. See the Supplementary Appendix for additional detail.

### Immunogenicity Analyses

Humoral immune responses were assessed by characterizing SARS-CoV-2 spike protein subunit (S) 1- and receptor-binding domain (RBD)–binding immunoglobulin G (IgG) concentrations, SARS-CoV-2 50% neutralizing titers (VN_50_) and 50% pseudovirus neutralizing titers (pVN_50_) in serum, and T-cell immune responses were characterized using *ex vivo* interferon gamma (IFN-γ) ELISpot and flow cytometry–based assays [15, 17–20]. For detailed experimental methods, see the Supplementary Appendix.

## RESULTS

### Analyzed Clinical Trials

#### BNT162-02

**Figure 1.**
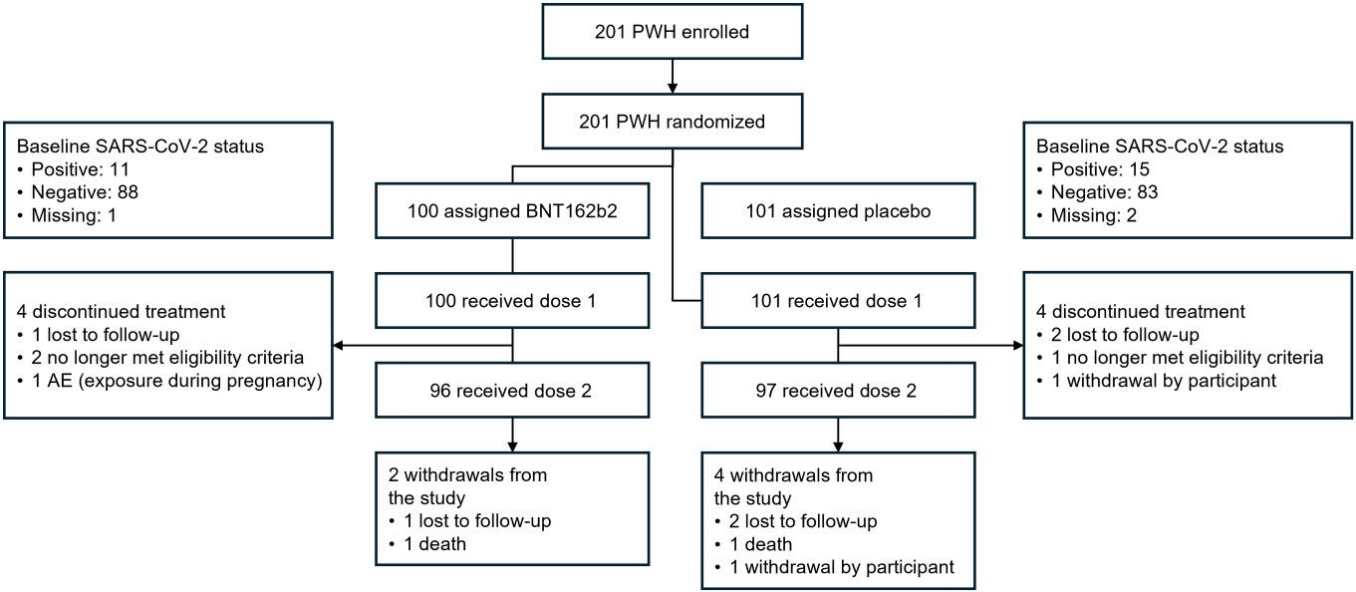
Participant disposition flow diagram for the BNT162-02 trial. At the time the safety analysis was performed for PWH (data cut-off date 13 March 2021), there were 200 participants identified as living with HIV, and the study was still ongoing. The immunogenicity analysis was performed using the final released database, in which 201 participants were identified as living with HIV as a result of an update to the medical history of 1 participant. A total of 201 PWH were randomized to receive 2 doses of 30 µg BNT162b2 (n = 100) or placebo (n = 101). In both groups, all participants received the first dose and 96 and 97 received the assigned second dose of BNT162b2 and placebo, respectively. Four participants discontinued treatment in each group. Two and 4 participants withdrew from the study in the BNT162b2 and placebo groups, respectively. Abbreviations: AE, adverse event; HIV, human immunodeficiency virus; PWH, people with human immunodeficiency virus; SARS-CoV-2, severe acute respiratory syndrome coronavirus 2.

BNT162-02 recruited 201 PWH between September and November 2020, in Argentina, Brazil, Germany, South Africa, Turkey, and the United States. A total of 100 PWH received BNT162b2 and 101 received placebo (primary 2-dose series spaced 21 days apart). All participants in both groups received dose 1, and 96 and 97 received dose 2 of BNT162b2 and placebo, respectively. Four participants from the BNT162b2 group withdrew from the trial between the first and second dose, and 2 BNT162b2 recipients withdrew during follow-up (Figure 1).

The vaccine and placebo groups were well matched in terms of sex and age (Supplementary Table 1). Both groups included 39% individuals with BMI ≥30.0 kg/m^2^. The majority (86%) of participants were SARS-CoV-2 negative; those positive in each group pre–dose 1 had a positive N-binding antibody result or a positive nucleic acid amplification test, or a medical history of COVID-19.

#### BNT162-01

BNT162-01 recruited 15 PWH at a single site in Germany, between February and March 2021. The HIV-negative cohort were recruited June to September 2020; data from participants aged 18–55 years in this cohort have been published previously [15]. All 15 PWH completed the vaccination schedule, and all protocol-specified visits and assessments. HIV-negative participants were matched as closely as possible by sex and age, and received the same 30-µg 2-dose primary dosing series as PWH. The groups were balanced with regard to sex, while the PWH group were on average slightly younger and of lower BMI than the control group (Supplementary Table 2). All participants were SARS-CoV-2 negative at baseline.

### Safety and Reactogenicity Following 2 Doses of BNT162b2 in PWH Were Comparable to Those Previously Reported in the Wider Population

#### BNT162-02

**Figure 2.**
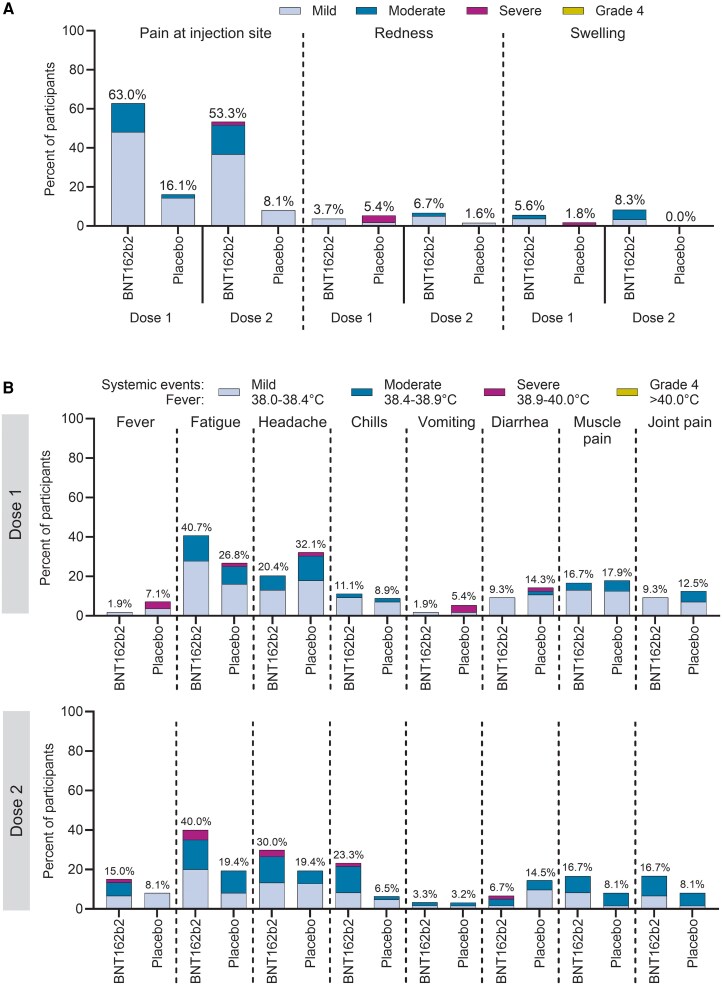
Solicited local reactions (*A*) and systemic events (*B*), by maximum severity, occurring within 7 days of each dose administered in people with human immunodeficiency virus (PWH) in the BNT162-02 trial. Local and systemic reactogenicity events were assessed in 146 PWH who received any dose of BNT162b2 (n = 72) or placebo (n = 74) and recorded at least 1 “yes” or “no” response for the specified event after the specific dose in their e-diary (dose 1, n = 110; dose 2, n = 122). Reactogenicity events were recorded in e-diaries for 7 days after each vaccination. Numbers above the bars are the total percent of participants who reported the specified reaction. Events were graded according to the US Food and Drug Administration Center for Biologics Evaluation and Research guidelines on toxicity grading scales for healthy adult volunteers (see Supplementary Methods for more information).

Safety was assessed in all vaccinated PWH and reactogenicity was analyzed in participants who submitted their e-diary as per protocol. Overall, higher rates of local reactions were reported by BNT162b2 recipients than placebo recipients. Most reactions were mild or moderate in severity with no grade 4 local or systemic reactions reported (Figure 2*A*). Among BNT162b2 recipients, pain at the injection site within 7 days of administration was the most common local reaction (63% vs 16% for placebo and 53% vs 8% following dose 1 and 2, respectively). Pain was typically mild or moderate, with <2% of BNT162b2 recipients reporting severe pain following the second injection and none reporting severe pain following the first. Rates of redness and swelling were low after both doses. The severity and frequency of local reactions reported by PWH were largely consistent with those reported from the reactogenicity subset in the phase 2/3 portion of the BNT162-02 trial, which did not include PWH [6].

Systemic reactogenicity events were reported more often by BNT162b2 recipients than placebo recipients (Figure 2*B*). The most common systemic events were fatigue, headache, muscle pain, and chills. While no BNT162b2 recipients reported a severe systemic event after the first dose, 5% reported severe fatigue and 3% reported severe headache after second dose. Severe fever (temperature, ≥38.9°C–40.0°C), severe chills, and severe diarrhea were each reported by 2% of BNT162b2 recipients after dose 2. Systemic events generally occurred at frequencies comparable or lower to those reported in the full phase 2/3 safety set [6], and resolved within 1–3 days.

Regarding unsolicited AEs, from dose 1 through 1 month post–dose 2 (D2 + 1m), a higher proportion of BNT162b2 recipients reported any AEs (26% and 13%, respectively) or AEs assessed as related to BNT162b2 per the investigator (19% and 3%) than placebo recipients (Supplementary Table 3). As with the full phase 2/3 safety set, reactogenicity events were reported as related AEs more commonly by BNT162b2 recipients than by placebo recipients. Frequencies of AEs reported by PWH who received BNT162b2 were similar to those reported in the full phase 2/3 safety set [6]. No SAEs assessed by investigators as related to BNT162b2 were reported up to D2 + 1m. The 1 withdrawal due to an AE was unrelated to BNT162b2 (exposure during pregnancy). There were no suspected unexpected serious adverse reactions. One participant died in each group; these deaths were assessed as not related to the trial treatment.

AEs were also reported as incidence rates (IRs; event/total exposure time in 100 person-years [PY]), to normalize for different durations of follow-up. Up until unblinding, the trend of unsolicited AE and SAE IRs was similar to that reported for the proportion of participants experiencing AEs/SAEs (Supplementary Table 4). IRs for (related) AEs were higher for BNT162b2 than placebo recipients, again due to reactogenicity events, at 62.8 and 10.4 IR/PY for PWH receiving BNT162b2 and placebo, respectively. SAE IRs were similar in the BNT162b2 and placebo groups (6.6 and 6.9 IR/PY, respectively), with no SAEs assessed as related to BNT162b2 in either group. Rates of study withdrawal due to AEs were higher for participants receiving BNT162b2 than placebo (6.6 vs 3.5 IR/PY for placebo).

#### BNT162-01

In the 15 PWH recruited to BNT162-01, local and systemic reactions up to 7 days post–dose 1 and dose 2 were comparable to those reported by the HIV-negative cohort (Supplementary Figures 1 and 2), and frequencies of AEs reported by PWH were comparable to those previously published in the wider HIV-negative population (Supplementary Table 5) [6, 15].

Safety profiles reported in both trials were generally comparable to those reported in the pivotal trial [6, 15].

### Immunogenicity Profiles Following 1 and 2 Doses of BNT162b2 in PWH Were Comparable to Those Reported in the Wider Population

#### BNT162-02

A total of 85 and 89 participants from the BNT162b2 and placebo groups were included in the dose 2 evaluable immunogenicity population, with 79 and 5 completing the 6-month post–dose 2 visit (participants in the placebo group crossed over to receive BNT162b2 following unblinding and demonstration of efficacy).

**Figure 3.**
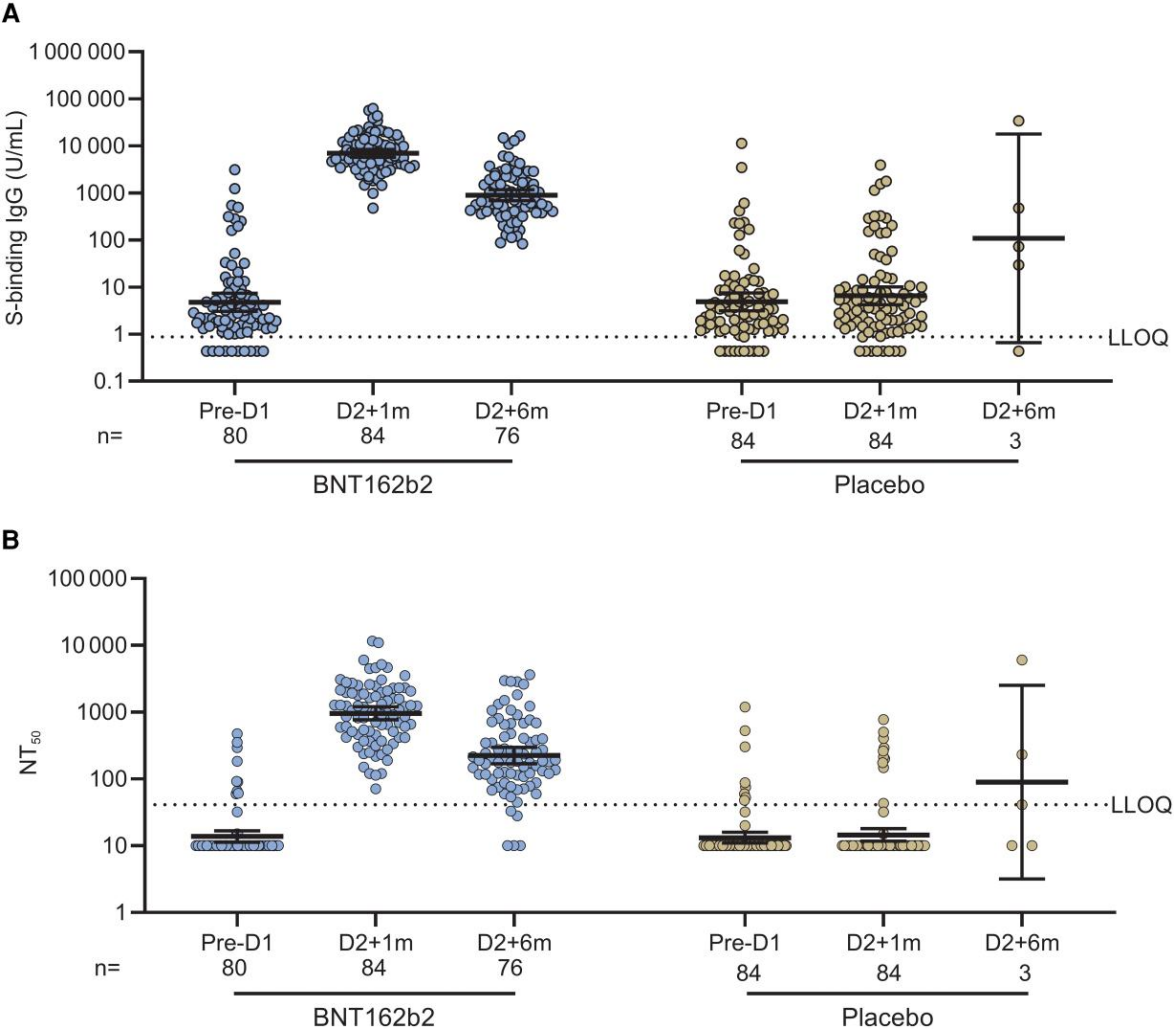
BNT162b2-induced virus binding (*A*) and neutralizing antibody (*B*) titers in people with human immunodeficiency virus (PWH) in the BNT162-02 trial. PWH in BNT162-02 were administered 2 doses of 30 µg BNT162b2 or placebo approximately 21 days apart. Serum samples were obtained pre–dose 1 (pre-D1), 1 month post–dose 2 (D2 + 1m), and 6 months post–dose 2 (D2 + 6m) and assessed for (*A*) full-length S-binding IgG titers and (*B*) NT_50_. Each data point represents a separate serum sample, and the bars represent the geometric mean concentrations (S-binding IgG) and geometric mean titers (NT_50_) with the 95% confidence intervals. Dashed lines indicate LLOQ: 0.873 and 41 for S-binding IgG and NT_50_, respectively. Results below the LLOQ are set to 0.5× LLOQ. Abbreviations: IgG, immunoglobulin G; LLOQ, lower limit of quantification; NT_50_, 50% neutralizing antibody titer; PWH, people with human immunodeficiency virus; S, spike protein.

In PWH who received BNT162b2, full-length S-binding IgG geometric mean concentrations (GMCs) increased substantially from pre-D1 to D2 + 1m, before decreasing by D2 + 6m (4.8, 6809.8, and 847.8 U/mL, respectively). In the placebo group, S-binding IgG GMCs were comparable between pre-D1 and D2 + 1m (Figure 3*A*, Supplementary Table 6). A similar trend was observed for SARS-CoV-2 VN_50_ geometric mean titers (GMTs) in BNT162b2 PWH recipients, with an increase observed by D2 + 1m from baseline that decreased by D2 + 6m (25.4, 939.7, and 216.2, respectively); mean titers were comparable between pre-D1 and D2 + 1m in the placebo group (Figure 3*B*, Supplementary Table 6). The detection of binding IgGs and neutralizing titers in a small number of participants in the placebo group may have been due to concurrent SARS-CoV-2 infection.

#### BNT162-01

**Figure 4.**
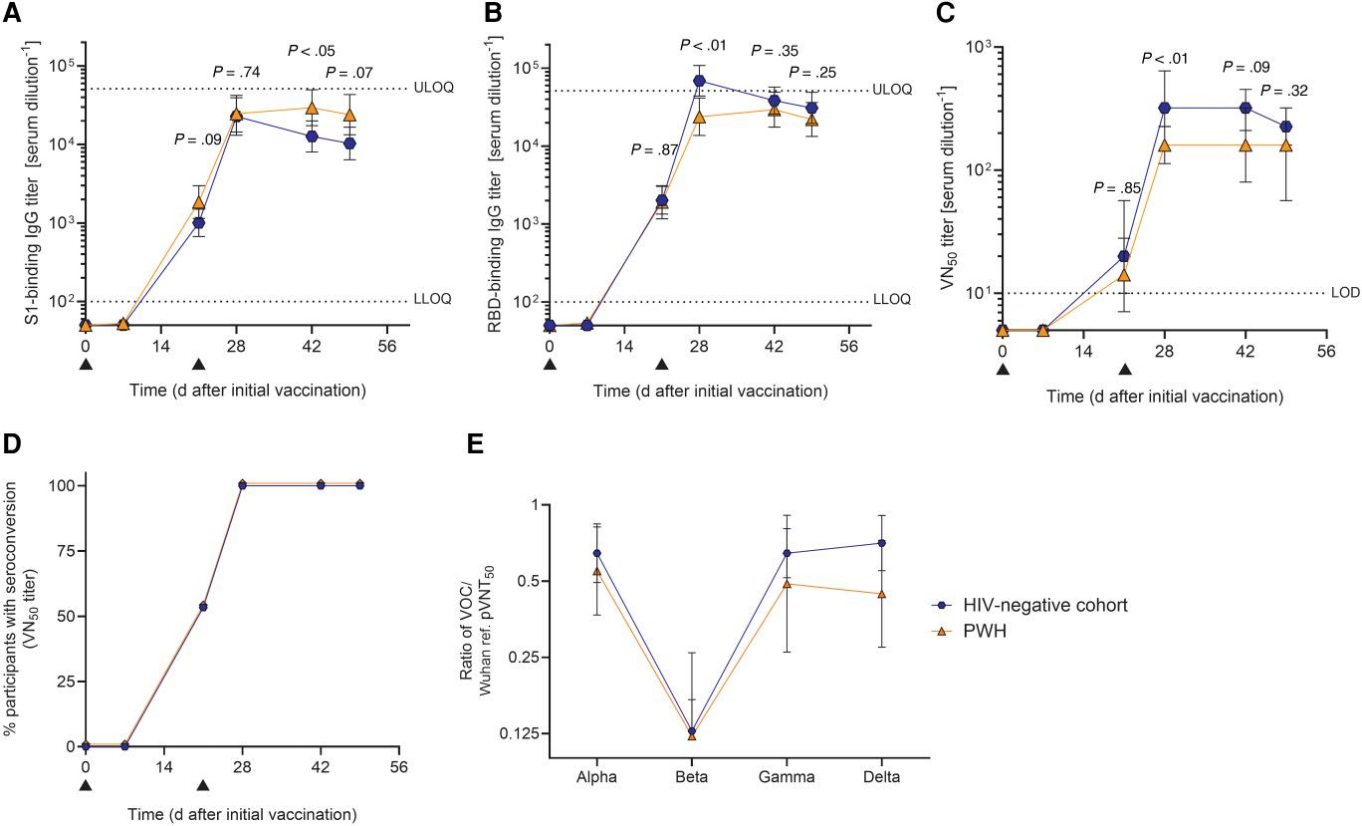
BNT162b2-induced S1- and RBD-binding and neutralizing antibody titers and seroconversion rates in the BNT162-01 trial. Participants from the BNT162-01 trial were immunized with 30 µg BNT162b2 on days 0 and 21 (n = 15 per cohort). Arrowheads indicate days of vaccination. GMTs (95% CI) for (*A*) recombinant S1- and (*B*) RBD-binding titers at each time point. Values below the LLOQ are set to 0.5× LLOQ. Values greater than the ULOQ are plotted as 2*ULOQ. The dotted horizontal lines represent the LLOQ and ULOQ. (*C)* GMTs (95% CI) for SARS-CoV-2 VN_50_ titers at each time point. Values smaller than the LOD are plotted as 0.5*LOD. The dotted horizontal line shows the LOD. (*D)* The frequency of participants with SARS-CoV-2 neutralizing GMT seroconversion at each time point post–dose 1. Seroconversion is defined as a minimum of a 4-fold increase of VN_50_ GMT compared to baseline. (*E)* Ratio of SARS-CoV-2 pVNT_50_ titers between VOC and wild-type reference strain spike pseudovirus. Group geometric mean pVN_50_ ratio with 95% CIs (from Supplementary Figure 2) at each time point are shown. Statistics were calculated using a nonparametric repeated Mann–Whitney test. Abbreviations: CI, confidence interval; d, day; GMT, geometric mean titer; HIV, human immunodeficiency virus; IgG, immunoglobulin G; LLOQ, lower limit of quantification; LOD, limit of detection; pVNT_50_, pseudovirus 50% neutralization; PWH, people with human immunodeficiency virus; RBD, receptor-binding domain; S1, spike protein 1; SARS-CoV-2, severe acute respiratory syndrome coronavirus 2; ULOQ, upper limit of quantification; VN_50_; 50% neutralization; VOC, variant of concern.

The general kinetics of the binding IgG responses against the N-terminal S1 domain and the RBD of the SARS-CoV-2 S protein were comparable between the PWH and HIV-negative groups and all reached levels close to the upper limit of quantification (ULOQ) around D2 + 7d (Figure 4*A* and 4*B*). An increase in binding IgG GMTs at D1 + 21d was followed by a further increase after dose 2. At D2 + 7d 11 of 15 HIV-negative participants displayed RBD-binding IgG GMTs above ULOQ of the RBD-binding enzyme-linked immunosorbent assay.

The general kinetics of the neutralizing antibody response were comparable between the PWH and HIV-negative groups, with a modest increase in SARS-CoV-2 neutralizing GMTs at D1 + 21d, and a substantial increase in GMTs after dose 2 (Figure 4*C*). On D2 + 7d and D2 + 21d, neutralizing GMTs tended to be lower in the PWH group (2.14-fold on D2 + 7d) than the HIV-negative group. Rates of SARS-CoV-2 neutralizing GMT seroconversion did not differ between groups, with around half seroconverting by D1 + 21d (day of dose 2 of BNT162b2); all participants had seroconverted by D2 + 7d and remained seropositive through D2 + 28d and beyond (Figure 4*D*).

The breadth of inhibition of variant virus entry by BNT162b2-elicited antibodies was investigated using a vesicular stomatitis virus–based SARS-CoV-2 pseudovirus neutralization assay (Figure 4*E*). No significant difference in the breadth of neutralization was observed between the PWH and HIV-negative groups, although neutralization of the Gamma (P1) and Delta (B.1.617.2) variants was lower in PWH compared with the HIV-negative participants.

### T-Cell Responses Elicited Following 2 Doses of BNT162b2 Were Comparable Between PWH and HIV-Negative Participants

Cell-mediated immune responses were evaluated using ELISpot and flow cytometry–based assays in participants with sufficient peripheral blood mononuclear cell (PBMC) samples (14 and 15 PWH, respectively, and 13 HIV-negative participants for both).

**Figure 5.**
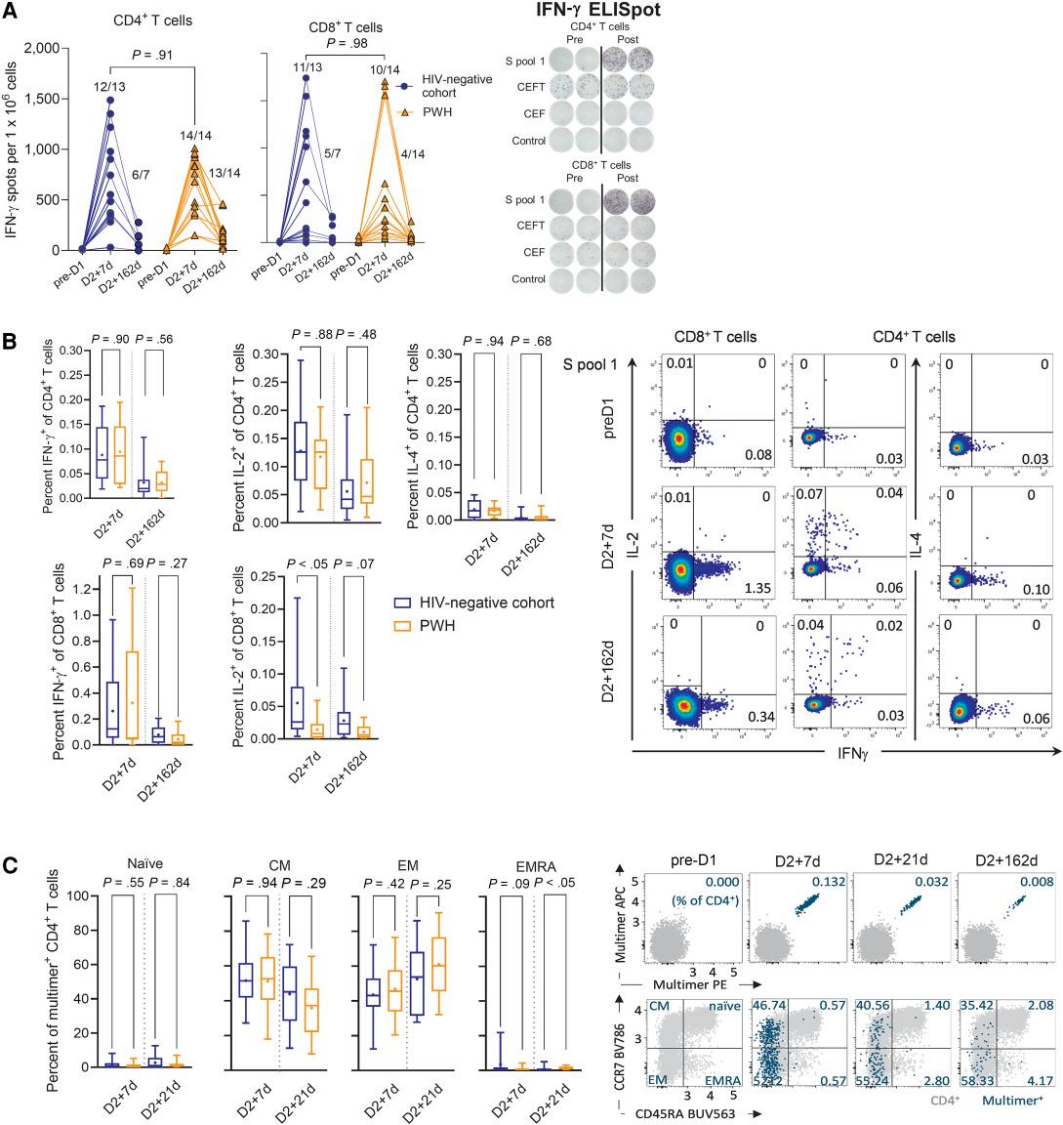
BNT162b2-induced CD4^+^ and CD8^+^ T-cell responses from PWH participants in the BNT162-01 trial. Participants were immunized with 30 µg BNT162b2 on days 0 and 21. (*A)* Left panels: PBMCs obtained from PWH (n = 14) and HIV-negative participants (n = 13) on days 0 (pre–dose 1 [pre-D1]), 28 (7 days post–dose 2 [D2 + 7d]), and 183 (162 days post–dose 2 [D2 + 162d]) were enriched for CD8^+^ and CD4^+^ T cells and analyzed using ex vivo ELISpot assay to assess the magnitude and durability of BNT162b2-induced T-cell responses. Each dot represents the normalized mean spot counts from duplicate wells stimulated with a peptide pool representing the N-terminal half of the wild-type SARS-CoV-2 S protein (peptide pool Sp1) for 1 participant, after subtraction of the medium-only control. CD4^+^ (left) and CD8^+^ (right) T-cell response data are shown. Ratios above postvaccination data points are the number of participants with detectable CD4^+^ or CD8^+^ T-cell responses within the total number of tested participants per cohort (responses compared with pre-D1). CD4^+^ spot count data from 1 participant from the HIV-positive cohort could not be background subtracted and normalized and hence was not included. Right panels: Example IFN-γ ELISpot assay from a single PWH participant, showing CD8^+^ and CD4^+^ T cells from prevaccination or postvaccination (day 28) that were stimulated overnight with Sp1; the positive controls CEFT and CEF, which contain cytomegalovirus, Epstein–Barr virus, and influenza virus (and tetanus toxoid for CEFT); and a negative media-only control. (*B)* The cytokine polarization and reactivities of BNT162b2-induced CD4^+^ and CD8^+^ T cells were characterized by ICS with flow cytometry, where PBMCs were stimulated overnight with Sp1, and analyzed by flow cytometry. T-cell cytokine data are plotted for HIV-negative participants (n = 13) and PWH (n = 15) from D2 + 7d and D2 + 162d in response to Sp1 peptide stimulation (left panels). Participant PBMCs were tested without replicates. For CD8^+^ T-cell analysis, 2 participants from the PWH group were not included due to a preexisting response against Sp1. Two participants from the PWH group were excluded due to high background in CD8^+^. The right panels show pseudocolor flow cytometry plots of cytokine-producing CD4^+^ and CD8^+^ T cells in response to Sp1 from 1 PWH participant. (*C)* Left panels: Relative frequencies of epitope-specific naive, CM, EM, and EMRA CD4^+^ T cells were determined by pMHC class II multimer staining (percent multimer positive of CD4^+^ T cells) using CCR7 and CD45RA markers, using PBMCs obtained on D2 + 7d and 21 days post–dose 2 (D2 + 21d). Right panels: Pseudocolor flow cytometry plots of frequencies of multimer-positive CD4^+^ T cells in response to Sp1 (upper row) from 1 PWH participant, assessed at pre-D1, D2 + 7d, D2 + 21d, and D2 + 162d. The lower row shows CD4^+^ T cells gated on naive, CM, EM, and EMRA populations at these time points. The box and whisker plots shown in (*B*) and (*C*) depict min-max, quartile, and median values; + indicates the mean. Statistics were calculated using a nonparametric Mann–Whitney test for ICS (*B*) and multimer (*C*) data. ELISpot data (*A*) were analyzed using subject response evaluation. All example assays were from a single participant. Abbreviations: APC, allophycocyanin; CEF, cytomegalovirus, Epstein–Barr virus, influenza virus; CEFT, CEF plus tetanus toxoid; CM, central memory; ELISpot, enzyme-linked immunosorbent spot assay; EM, effector memory; EMRA, terminally differentiated effector memory; HIV, human immunodeficiency virus; ICS, intracellular cytokine staining; IFN-γ, interferon gamma; PBMCs, peripheral blood mononuclear cells; PE, phycoerythrin; pMHC, peptide–major histocompatibility complex; PWH, people with human immunodeficiency virus; S protein, severe acute respiratory syndrome coronavirus 2 spike protein.

Using the ex vivo IFN-γ ELISpot assay, we quantified IFN-γ secretion by CD8^+^ and CD4^+^ T cells specific for the N-terminal portion of the S protein (Sp1 pool). At D2 + 7d, all PWH participants (14/14) displayed CD4^+^ T-cell responses, and 10 of 14 (71%) had detectable antigen-specific IFN-γ–secreting CD8^+^ cells, which was broadly comparable to those detected in the HIV-negative group (12/13 [92%] and 11/13 [85%] CD4^+^ and CD8^+^ responses, respectively). In PWH, CD4^+^ T-cell responses were detected up to D2 + 162d (13/14 [93%]), although the magnitude of responses declined. However, there was a reduced CD8^+^ T-cell response rate at D2 + 162d (4/14 [29%]; Figure 5*A*). Comparable RBD-specific CD8^+^ and CD4^+^ T-cell responses were seen in both groups on D2 + 7d (Supplementary Figure 3).

Longitudinal analysis of vaccine-induced CD4^+^ and CD8^+^ T-cell responses specific for Sp1 using intracellular cytokine staining revealed similar responses between PWH and HIV-negative participants at D2 + 7d and D2 + 162d, albeit with a reduced CD8^+^ interleukin (IL)-2 response in PWH versus HIV-negative participants at D2 + 7d (Figure 5*B*; Supplementary Figure 4). Similar CD4^+^/CD8^+^ T-cell cytokine profile trends were observed between groups with Sp1 pool, RBD, or Sp2 pool (represents the more conserved C-terminal end of S) peptide stimulation (Supplementary Figures 3 and 5), suggesting that T-cell responses were directed against epitopes distributed throughout the entire SARS-CoV-2 S protein. CD4^+^ T cells exhibited a Th1 polarization characterized by the production of IFN-γ and IL-2 and limited amounts of IL-4 (Figure 5*B*).

Fluorescently labeled peptide–major histocompatibility complex (pMHC) multimer cocktails with pMHC allele pairs were used to detect pMHC-binding (and therefore epitope-specific) CD4^+^ T-cell complexes by flow cytometry. At day 0, D2 + 7d, D2 + 21d, and D2 + 162d, we identified *de novo* multimer-positive CD4^+^ T-cell populations for every participant assayed (n = 10 PWH and n = 10 HIV-negative participants), representing 10 different epitope–MHC pairs, and revealed that multimer-positive cells peaked at D2 + 7d, with approximately 10-fold fewer cells detectable on D2 + 162d compared to D2 + 7d. As such, the most robust comparison of cell phenotypes could be made at D2 + 7d and D2 + 21d. Cell populations in both groups were predominantly central memory and effector memory (Figure 5*C*).

## DISCUSSION

Safety data demonstrate that two 30-µg doses of BNT162b2 were well tolerated in PWH with stable disease on ART. Local and systemic reactions following vaccination with BNT162b2 were largely consistent with those observed in HIV-negative participants in the phase 2/3 portion of the BNT162-02 trial [6]. Serological responses elicited by BNT162b2 in PWH in both trials were comparable to those previously reported in the wider population [5], including HIV-negative individuals [15]. Mildly lower neutralization titers were observed in PWH versus HIV-negative participants following the second vaccination. It is unclear if this may be related to ART interference in the detection of serum neutralizing antibodies, as reported previously [21]. Moreover, effective cross-neutralization against Alpha, Beta, Gamma, and Delta VOC pseudovirus from sera of PWH vaccinated with BNT162b2 is in line with previous findings [14].

In this study, vaccine-induced CD4^+^ and CD8^+^ T-cell responses were largely similar in PWH and HIV-negative participants at D2 + 7d, with all PWH developing T-cell responses. The observation that CD8^+^ IL-2^+^ T-cell responses in PWH were significantly lower than HIV-negative participants at D2 + 7d could be attributed to a functional impairment of CD8^+^ T cells under chronic inflammatory conditions, a mechanism described in the context of HIV infection and CD8^+^ T-cell responses to influenza vaccination [22–24]. T-cell responses induced by BNT162b2 in PWH appeared otherwise similar in terms of magnitude and cytokine profile to that seen in HIV-negative participants. While the evidence that BNT162b2 induces humoral and cellular responses presented here comes from a relatively small cohort, the results are consistent with other studies that have reported BNT162b2-induced immunogenicity in PWH on ART [8–10, 14, 25, 26].

The data presented here have limitations. A history of CD4^+^ T-cell counts, opportunistic infections during the study, time from HIV infection to diagnosis and ART, repeated viral load measures, and concomitant medication were not obtained. Furthermore, asymptomatic surveillance of participants was not conducted during the trial, meaning breakthrough SARS-CoV-2 infections may have been undetected. The PWH enrolled in both the BNT162-01 and BNT162-02 trials had stable HIV disease; therefore, the data cannot be generalized to those PWH who are undiagnosed, not on ART, or experiencing virological failure, or translated into efficacy. Further safety and immunogenicity data in individuals without stable HIV disease should be obtained given their weakened immune status.

In summary, a 2-dose BNT162b2 vaccination series in PWH on ART was well tolerated and immunogenic, supporting its use in this population. Owing to waning immunity and vaccine escape variants, additional vaccination with variant-adapted COVID-19 vaccines, which is known to provide greater immunity and protection against VOCs [20, 27], is recommended for those who are moderately or severely immunocompromised [4]. To design the most effective vaccination strategies for PWH and other vulnerable populations, future studies should characterize longer-term immunogenicity and the immunogenicity of additional vaccinations.

## Supplementary Material

ofag310_Supplementary_Data

## Supplementary Data

Supplementary materials are available at *Open Forum Infectious Diseases* online. Consisting of data provided by the authors to benefit the reader, the posted materials are not copyedited and are the sole responsibility of the authors, so questions or comments should be addressed to the corresponding author.
