# Supplementary material for: Safety and Immunogenicity of BNT162b2 in Antiretroviral Therapy–Treated People With HIV
Source: Open Forum Infect Dis. 2026 Jun 21;13(7):ofag310. doi: 10.1093/ofid/ofag310 (PMC13386175; doi:10.1093/ofid/ofag310)
Supplement: ofag310_Supplementary_Data [file ofag310_supplementary_data.docx]

# Supplementary materials for Mitha E, et al. 2026: Safety and immunogenicity of BNT162b2 in ART-treated PWH

# Supplementary Figures Supplementary Figure 1: Frequency of solicited local reactions (assessed by participant) by maximum severity, occurring within 7 days of Doses 1 and 2 in the BNT162-01 trial

HIV-neg, human immunodeficiency virus negative; PWH, people with human immunodeficiency virus.

Local reactogenicity events were assessed in 15 PWH and 15 HIV-negative controls who received any dose of BNT162b2. Reactogenicity events were recorded for 7 days after each vaccination. Numbers above the bars are the total percent of participants who reported the specified reaction. Events were graded according to FDA Center for Biologics Evaluation and Research (CBER) guidelines on toxicity grading scales for healthy adult volunteers, see supplemental methods for more information.

## Supplementary Figure 2: Frequency of solicited systemic reactions (assessed by participant) by worst grade following Doses 1 and 2 in the BNT162-01 trial

HIV-neg, human immunodeficiency virus negative; PWH, people with human immunodeficiency virus.

Systemic reactogenicity events were assessed in 15 PWH and 15 HIV-negative controls who received any dose of BNT162b2. Reactogenicity events were recorded for 7 days after each vaccination. Numbers above the bars are the total percent of participants who reported the specified reaction. Events were graded according to FDA Center for Biologics Evaluation and Research (CBER) guidelines on toxicity grading scales for healthy adult volunteers, see supplemental methods for more information.

## Supplementary Figure 3: BNT162b2-induced T-cell responses (RBD IFNγ ELISpot and ICS) in the BNT162-01 trial

ELISpot, enzyme-linked immunosorbent spot; ICS, intracellular cytokine staining; IFNγ, interferon gamma; PWH, people with human immunodeficiency virus; RBD, receptor-binding domain.

Participants were immunized with 30 µg BNT162b2 on Days 0 and 21. Peripheral blood mononuclear cells (PBMCs) obtained on Days 0 (pre-Dose 1; preD1), 28 (7 days post-Dose 2; D2+7d), and 183 (162 days post-Dose 2; D2+162d) were enriched for CD8+ and CD4^+^ cells and analyzed using *ex vivo* IFNγ ELISpot assay (A). Each dot represents the normalized mean spot counts from duplicate wells stimulated with an RBD peptide pool for one participant, after subtraction of the medium-only control. Ratios above post-vaccination data points are the number of participants with detectable CD4^+^ or CD8^+^ T-cell responses within the total number of tested participants per cohort. CD4^+^ spot count data from one participant from the PWH cohort could not be background subtracted and normalized and hence was not included. CD4^+^ (B) and CD8^+^ (C) T cell cytokine data are plotted for HIV-negative participants (n=13) and PWH (n=15) from Day 28 and n=12 for both cohorts from D2+162d in response to RBD peptide stimulation. Box-Whisker plots depict min/max values, and quartile and median values, + indicates the mean. For CD8^+^ analysis, two participants from the PWH group were not included due to a pre-existing response against RBD. Two participants from the PWH group were excluded due to high background in CD8^+^.

Statistics calculated using a non-parametric Mann-Whitney test.

## Supplementary Figure 4: BNT162b2-induced T-cell responses (Sp1 ICS assay) in the BNT162-01 trial

ICS, intracellular cytokine staining; IFNγ, interferon gamma; IL, interleukin; PWH, people with human immunodeficiency virus; Sp1, S protein peptide pool 1.

Participants were immunized with 30 µg BNT162b2 on Day 0 and 21. Vertical dashed lines indicate vaccination on Day 21. CD4^+^ (A) and CD8^+^ (B) cytokine data are plotted for HIV-negative participants (n=13, all timepoints) and PWH (n=15 Days 0 [pre-Dose 1], 28 [7 days after Dose 2], 42 [21 days after Dose 2]; n=13, Days 84 [63 days after Dose 2] and 183 [162 days after Dose 2] in response to Sp1 peptide stimulation. Samples were not available for one HIV-negative participant for D2+21d, D2+83d and D2+162d, and for a further HIV-negative participant for D2+21d. In addition, CD8^+^ data sets were excluded for two PWH due to high background in unstimulated samples.

## Supplementary Figure 5: BNT162b2-induced T-cell responses (Sp2 ICS assay) in the BNT162-01 trial

ICS, intracellular cytokine staining; IFNγ, interferon gamma; IL, interleukin; PWH, people with human immunodeficiency virus; Sp2, S protein peptide pool 2

Participants were immunized with 30 µg BNT162b2 on Days 0 and 21. Vertical dashed lines indicate vaccination on Day 21.

CD4^+^ (A) and CD8+ (B) cytokine data are plotted for HIV-negative participants (n=13, all timepoints) and PWH (n=15 Days 0 [pre-Dose 1], 28 [7 days after Dose 2], 42 [21 days after Dose 2]; n=13, Days 84 [63 days after Dose 2] and 183 [162 days after Dose 2] in response to Sp2 peptide stimulation. Samples were not available for one HIV-negative participant for D2+21d, D2+63d, and D2+162d, and for a further HIV-negative participant for D2+21d. CD8^+^ data from two PWH were excluded due to high background in unstimulated samples. In addition, non-specific CD8^+^ cytokine data are excluded for HIV-negative participants (n=4) and PWH (n=7).

## Supplementary Figure 6: Flow cytometry gating strategy for cytokine analysis by ICS


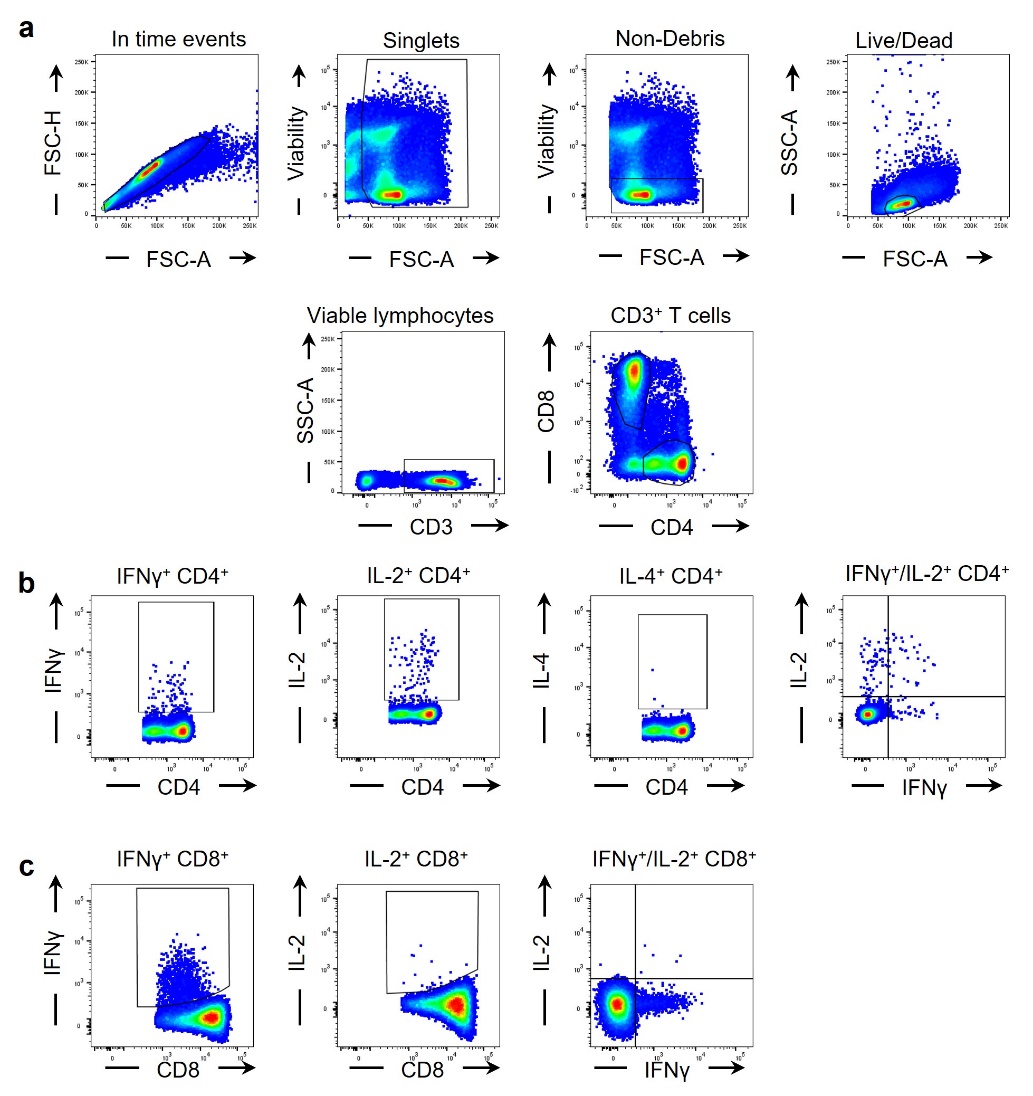


FSC-A, forward scatter area; FSC-H, forward scatter height; ICS, intracellular cytokine staining; IFNγ, interferon gamma; IL, interleukin.

Gating strategy for identification of IFNγ, IL-2 and IL-4 secreting T cells in peripheral blood mononuclear cell samples from an example PWH participant. a, CD4^+^ and CD8+ T cells were gated within single, viable lymphocytes. b, c, Gating of IFNγ, IL-2 and IL-4 in CD4^+^ T cells (b), and IFNγ and IL-2 in CD8+ T cells (c).

**Supplementary Figure 7:** Gating strategy for pMHC-class-II multimer staining by flow cytometry.


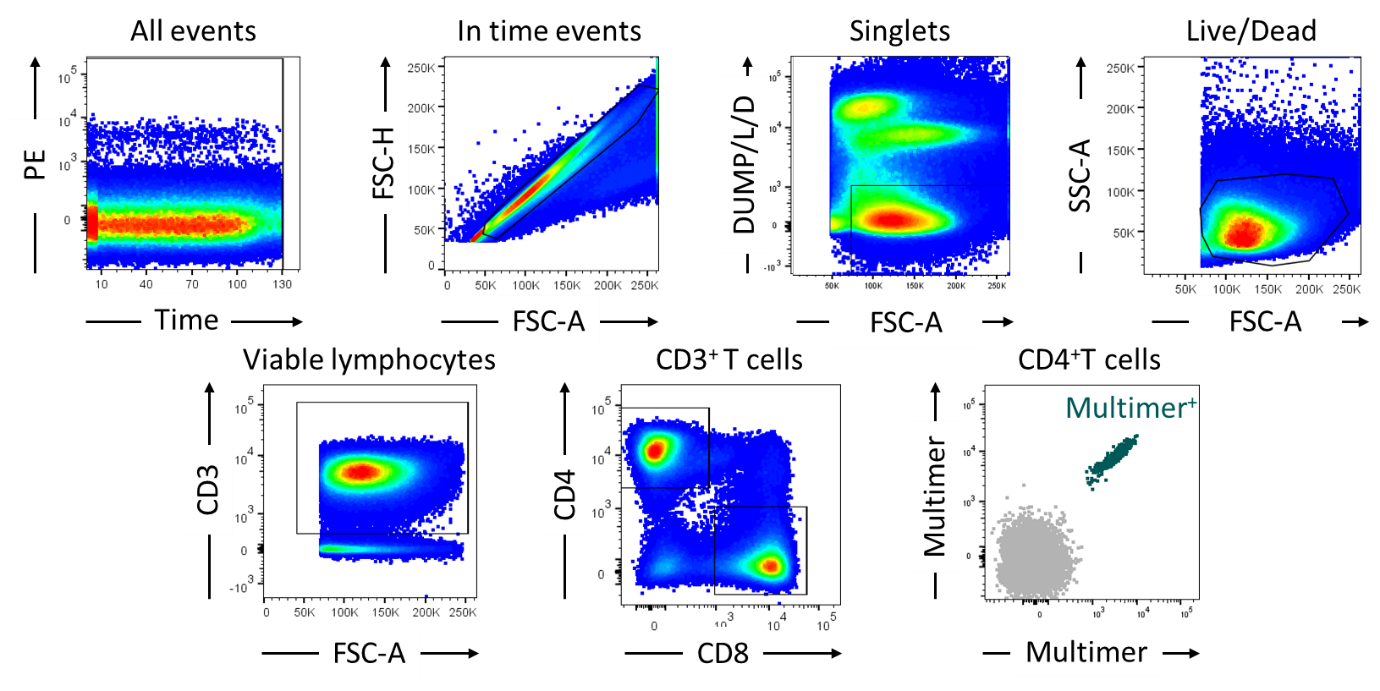


FSC-A, forward scatter area; FSC-H, forward scatter height; L/D, live/dead; PE, phycoerythrin; pMHC, peptide-major histocompatibility complex.

Gating strategy for identification and characterization of antigen-specific CD4^+^ T cells in peripheral blood mononuclear cell samples. Successive gates were applied to identify singlets, lymphocytes, DUMP (CD14, CD19, CD16 positive) and dead (L/D negative) cells, CD3^+^ T cells, and CD4^+^ or CD8^+^ T cells. Antigen-specific CD4^+^ T cells were gated as multimer double positive cells (green), with a combination of two fluorochromes labeling a defined MHC-epitope.

**Supplementary Figure 8:** pMHC-class-II multimers

 HLA, human leukocyte antigen; pMHC, peptide-major histocompatibility complex; RBD, receptor-binding domain; S, spike.

Genetic map spanning the length of the wild-type SARS-CoV-2 Spike sequence. Positive pMHC II-binding epitopes are marked in squares, with colors representing the HLA restrictions a position correlating to the binding location along the Spike sequence. Frequency percentages above correspond to the frequency of each allele and whether the allele-specific epitopes are located within the S1 or S2 portion of Spike. Total number of detected epitopes are used to calculate the S1 or S2 frequencies.

# Supplementary Tables

## Supplementary Table 1: Baseline characteristics by group in the PWH Cohort of the BNT162-02 trial

| Variable | BNT162b2 (N=100) | Placebo (N=100)^a^ |
| --- | --- | --- |
| Sex |  |  |
| Male | 69 (69.0) | 66 (66.0) |
| Female | 31 (31.0) | 34 (34.0) |
| Age group (at vaccination) |  |  |
| 16–55 Years | 74 (74.0) | 69 (69.0) |
| >55 Years | 26 (26.0) | 31 (31.0) |
| Age at vaccination (years) |  |  |
| Mean (SD) | 49.0 (9.74) | 48.9 (11.15) |
| Median | 50.0 | 49.0 |
| Min, max | (22, 75) | (26, 68) |
| Race |  |  |
| Black or African American | 52 (52.0) | 57 (57.0) |
| White | 44 (44.0) | 37 (37.0) |
| American Indian or Alaska Native | 1 (1.0) | 2 (2.0) |
| Asian | 2 (2.0) | 1 (1.0) |
| Multiracial | 1 (1.0) | 2 (2.0) |
| Not reported | .. | 1 (1.0) |
| Baseline SARS-CoV-2 status |  |  |
| Positive^b^ | 15 (15.0) | 11 (11.0) |
| Negative^c^ | 83 (83.0) | 88 (88.0) |
| Data missing | 2 (2.0) | 1 (1.0) |
| CD4^+^ cell count |  |  |
| <200 cells/mm^3, d^ | 2 (2.0) | 2 (2.0) |
| 200–500 cells/mm^3^ | 16 (16.0) | 28 (28.0) |
| >500 cells/mm^3^ | 78 (78.0) | 64 (64.0) |
| Data missing | 4 (4.0) | 6 (6.0) |
| Viral load |  |  |
| <50 copies/mL | 93 (93.0) | 96 (96.0) |
| ≥50 copies/mL^d^ | 4 (4.0) | 0 |
| Missing^d^ | 3 (3.0) | 4 (4.0) |
| BMI |  |  |
| <18.5 kg/m^2^ | 4 (4.0) | 1 (1.0) |
| ≥18.5 kg/m^2^–24.9 kg/m^2^ | 22 (22.0) | 26 (26.0) |
| ≥25.0 kg/m^2^–29.9 kg/m^2^ | 35 (35.0) | 34 (34.0) |
| ≥30.0 kg/m^2^ | 39 (39.0) | 39 (39.0) |

Data presented are n (%) unless otherwise stated.

ART, antiretroviral therapy; BMI, Body Mass Index; NAAT, nucleic acid amplification test.

^a^At the time the safety analysis was performed for PWH (data cut-off date 13 March 2021) there were 200 participants identified as living with HIV, and the study was still ongoing. The immunogenicity analysis was performed using the final released database, in which 201 participants were identified as living with HIV as a result of an update to the medical history of one participant.

^b^Positive N-binding antibody result at Visit 1, positive NAAT result at Visit 1, or medical history of COVID-19.

^c^Negative N-binding antibody result at Visit 1, negative NAAT result at Visit 1, and no medical history of COVID-19.

^d^Although not originally recorded as protocol deviations, enrollment of individuals with CD4^+^ cell count and viral loads outside of the stated protocol enrollment criteria were later identified as deviations.

## Supplementary Table 2: Baseline characteristics by group in the BNT162-01 trial

| Variable | PWH (N=15) | HIV-negative (N=15) |
| --- | --- | --- |
| Sex |  |  |
| Male | 12 (80) | 11 (73) |
| Female | 3 (20) | 4 (27) |
| Age (years) | 45.3 (36.2–70.1) | 49.9 (35.8–68.2) |
| Race |  |  |
| White | 14 (93) | 15 (100) |
| Asian | 1 (7) | .. |
| On ART | 15 (100) | .. |
| BMI (kg/m^2^) | 23.3 (21.0–27.5) | 25.2 (22.8–28.1) |

Data presented are n (%) or median (range).

ART, antiretroviral therapy; BMI, Body Mass Index.

# Supplementary Table 3: Safety overview from Dose 1 to one-month post-Dose 2 for PWH ≥16 years of age from BNT162-02

| Variable | BNT162b2 (N=100) | Placebo (N=100) |
| --- | --- | --- |
| Any AE | 26 (26) | 13 (13) |
| Any related AE | 19 (19) | 3 (3) |
| Any SAE | 0 | 0 |
| Any related SAE^a^ | 0 | 0 |
| Withdrawal due to AEs^b^ | 1 (1) | 0 |
| Death | 0 | 0 |
| AEs reported in ≥5% of participants | | |
| Injection site pain | 11 (11) | 0 |
| Fatigue | 7 (7) | 1 (1) |
| Pyrexia | 7 (7) | 0 |
| Chills | 6 (6) | 1 (1) |
| Myalgia | 6 (6) | 2 (2) |

Data are reported are n (%).
AE, adverse event; PWH, people with human immunodeficiency virus; SAE, serious adverse event
^a^Per the investigator’s assessment

^b^Exposure during pregnancy

# Supplementary Table 4: Incidence rates of adverse events From Dose 1 to unblinding for PWH ≥ 16 years of age from BNT162-02

| Variable | BNT162b2 (N=100) | Placebo (N=100) |
| --- | --- | --- |
| Any AE | 95.8 | 52.0 |
| Any related AE^a^ | 62.8 | 10.4 |
| Any SAE | 6.6 | 6.9 |
| Any related SAE^a^ | 0 | 0 |
| Withdrawal due to AEs | 6.6 | 3.5 |
| Death | 3.3 | 3.5 |

^a^Per the investigator’s assessment

AE, adverse event; PWH, people with human immunodeficiency virus; SAE, serious adverse event.

Incidence rates of AEs and SAEs, calculated as number of participants reporting the event/total exposure time in 100 person-years (PY) across all participants in the specified group.

# Supplementary Table 5: Safety overview from Dose 1 to one month post-Dose 2 for PWH ≥18 years of age from BNT162-01

| Variable | PWH (N=15) |
| --- | --- |
| Any TEAE | 3 (20) 7 |
| Any related TEAE^a^ | 2 (13) 4 |
| Any Grade ≥3 TEAE | 0 |
| Any TESAE | 0 |
| Any TEAE resulting in death | 0 |
| TEAEs reported in ≥5% of participants | |
| Myalgia | 2 (13) |
| Cellulitis | 1 (7) |
| Oral herpes | 1 (7) |
| Back pain | 1 (7) |
| Malaise | 1 (7) |
| Musculoskeletal stiffness | 1 (7) |

Data are reported are n (%) E (number of events).
PWH, people with human immunodeficiency virus; SAE, serious adverse event; TEAE, treatment-emergent adverse event; TESAE, treatment-emergent serious adverse event.
^a^Per the investigator’s assessment

# Supplementary Table 6: Geometric mean concentrations (S-binding IgG) and geometric mean titers (NT50) by baseline SARS-CoV-2 status in the BNT162-02 trial

|  |  |  | BNT162b2 | | | Placebo | | | |
| --- | --- | --- | --- | --- | --- | --- | --- | --- | --- |
|  |  |  | All | Positive | Negative | | All | Positive | Negative |
| S-binding IgG | PreD1 | n/m | 80/91 | 10/11 | 70/80 | | 84/90 | 7/7 | 77/83 |
|  |  | GMC (95% CI) | 4.8 (3.1, 7.4) | 360.6 (161.3, 806.4) | 2.6 (2.0, 3.4) | | 4.9 (3.1, 7.5) | 129.1 (18.0, 923.9) | 3.6 (2.4, 5.4) |
|  | D2+1m | n/m | 84/92 | 12/13 | 70/77 | | 84/95 | 6/10 | 78/85 |
|  |  | GMC (95% CI) | 6809.8 (5602.9, 8276.7) | 21443.6 (13686.0, 33598.3) | 5656.3 (4685.2, 6828.6) | | 5.0 (3.4, 7.3) | 236.4 (173.5, 322.1) | 3.7 (2.6, 5.1) |
|  | D2+6m | n/m | 76/89 | 11/13 | 63/74 | | 3/6 | 0/2 | 3/4 |
|  |  | GMC (95% CI) | 847.8 (652.0, 1102.4) | 2991.0 (1480.4, 6043.2) | 677.9 (521.4, 881.2) | | 18.3 (0.0, 115604.7) | NE | 18.3 (0.0, 115604.7) |
| NT50 | PreD1 | n/m | 80/91 | 10/11 | 70/80 | | 84/90 | 7/7 | 77/83 |
|  |  | GMT (95% CI) | 25.4 (21.9, 29.4) | 85.8 (41.2, 178.7) | 21.3 (19.7, 23.2) | | 24.7 (21.3, 28.6) | 45.8 (26.6, 78.7) | 23.3 (20.1, 27.1) |
|  | D2+1m | n/m | 84/92 | 12/13 | 70/77 | | 84/95 | 6/10 | 78/85 |
|  |  | GMT (95% CI) | 939.7 (750.8, 1176.1) | 4082.2 (2815.1, 5919.6) | 727.1 (589.1, 897.5) | | 24.1 (21.0, 27.6) | 106.6 (36.0, 315.6) | 21.5 (19.6, 23.6) |
|  | D2+6m | n/m | 76/89 | 11/13 | 63/74 | | 3/6 | 0/2 | 3/4 |
|  |  | GMT (95% CI) | 216.2 (164.3, 284.4) | 1012.7 (578.8, 1771.9) | 166.1 (127.0, 217.1) | | 46.0 (1.4, 1493.8) | NE | 46.0 (1.4, 1493.8) |

Data reported in the top row of each timepoint are n/m where n = number of participants with evaluable samples and m = all available immunogenicity population.

CI, confidence interval; D, dose; GMC, geometric mean concentration; GMT, geometric mean titer; IgG, immunoglobulin G; m, month; NE, not evaluable; NT50, 50% neutralizing titer; S, spike protein

## Supplementary Table 7: Overview of mutations per VOC SARS-CoV-2 S sequence

| SARS-CoV-2 VOC | Mutations in the S protein |
| --- | --- |
| Alpha | ΔH69, ΔV70, ΔY144, N501Y, A570D, D614G, P681H, T716I, S982A, D1118H |
| Beta | L18F, D80A, D215G, ΔL242, ΔA243, ΔL244, R246I, K417N, E484K, N501Y, D614G, A701V |
| Gamma | L18F, T20N, P26S, D138Y, R190S, K417T, E484K, N501Y, H655Y, T1027I, V1176F |
| Delta | T19R, G142D, ΔF157, ΔR158, L452R, T478K, D614G, P681R, D950N, K986P, V987P |

S, spike; VOC, variant of concern.

# Supplementary Methods

## Randomization/Allocation and Masking

## *BNT162-02*

PWH in the BNT162-02 trial were randomized using an interactive response technology system (IWR). Trial and site personnel, including the investigator, investigator staff, and participants, were blinded to trial intervention assignments except for an unblinded dispenser/administrator. In particular, the individuals who evaluate participant safety were blinded.

*BNT162-01*

BNT162-01 was a non-randomized, non-blinded study. As such, trial participants were not assigned to the trial dose groups according to a randomization plan but were assigned to the expansion cohort for immunocompromised individuals according to age (younger versus older participants) and HIV status.

## Clinical Procedures

## *BNT162-02*

At the initial visit, a nasal (midturbinate) swab was collected to test participants for SARS-CoV-2 using nucleic acid amplification testing. A clinical assessment and, when indicated, a physical examination was performed.

Pain at the injection site was assessed according to the following scale: mild, does not interfere with activity; moderate, interferes with activity; severe, prevents daily activity; and Grade 4, emergency department visit or hospitalization. Redness and swelling were measured according to the following scale: mild, 2.0 to 5.0 cm in diameter; moderate, >5.0 to 10.0 cm in diameter; severe, >10.0 cm in diameter; and Grade 4, necrosis or exfoliative dermatitis (for redness) and necrosis (for swelling). For systematic reactions, headache, fatigue, chills, new or worsened muscle pain and new or worsened joint pain were graded according to: mild, does not interfere with activity; moderate, some interference with activity; or severe, prevents daily activity. Vomiting was graded according to: mild, 1 to 2 times in 24 hours; moderate, >2 times in 24 hours; or severe, requires intravenous hydration. Diarrhea was graded according to: mild, 2 to 3 loose stools in 24 hours; moderate, 4 to 5 loose stools in 24 hours; or severe, 6 or more loose stools in 24 hours. Grade 4 for all events indicated an emergency department visit or hospitalization. Fever was graded according to: mild, ≥38.0 to 38.4°C; moderate, >38.4 to 38.9°C; severe, >38.9 to 40.0°C; Grade 4 >40.0°C.

The safety population included randomized participants who had received ≥1 dose of study intervention. Demographics and safety data are available for N=200 participants aged 16 to 85 with stable HIV infection and suppressed viral load.

*BNT162-01*

At the initial visit, blood samples were collected to test participants for SARS-CoV-2 and anti- SARS-CoV-2 antibodies. PWH were tested locally for CD4^+^ T cell count. A clinical assessment and when indicated, a physical examination was performed.

Pain at the injection site was assessed according to the following scale: mild, does not interfere with activity; moderate, interferes with activity; severe, prevents daily activity; and Grade 4, emergency department visit or hospitalization. Tenderness was assessed according to the following scale: mild, mild discomfort to touch; moderate, discomfort with movement; severe, significant discomfort at rest; and Grade 4, emergency department visit or hospitalization. Redness and swelling were measured according to the following scale: mild, 2.5 to 5.0 cm in diameter; moderate, >5.0 to 10.0 cm in diameter; severe, >10.0 cm in diameter; and Grade 4, necrosis or exfoliative dermatitis (for redness) and necrosis (for swelling). For systematic reactions, headache, fatigue, chills, new or worsened muscle pain and new or worsened joint pain were graded according to: mild, does not interfere with activity; moderate, some interference with activity; or severe, prevents daily activity. Vomiting was graded according to: mild, 1 to 2 times in 24 hours; moderate, >2 times in 24 hours; or severe, requires intravenous hydration. Diarrhea was graded according to: mild, 2 to 3 loose stools in 24 hours; moderate, 4 to 5 loose stools in 24 hours; or severe, 6 or more loose stools in 24 hours. Grade 4 for all indicated an emergency department visit or hospitalization. Fever was graded according to: mild, 38.0 to 38.4°C; moderate, 38.5 to 38.9°C; severe, 39.0 to 40.0°C; Grade 4 >40.0°C.

Demographics and safety data are available for 15 PWH and 15 HIV-negative control participants. Data showing AEs and SAEs from BNT162-01 are presented up to 28 days post-Dose 2.

BNT162b2 is formulated in lipid nanoparticles (Acuitas Therapeutics Inc, Vancouver, Canada) and has been previously described in Walsh et al. 2020 and Sahin et al. 2021[5, 15]. In both studies, PWH were vaccinated with a primary two-dose series of BNT162b2 (30 μg) or saline placebo administered intramuscularly 21 ± 2 days apart into the deltoid muscle. Following dosing, participants were observed for 30 min to identify immediate AEs.

## Laboratory Procedures: BNT162-02

### Immunogenicity assays

Serum from participants was collected and isolated from whole blood via centrifugation at trial sites, shipped on dry ice, and thawed prior to use in immunogenicity assays. Anti-SARS-CoV-2 specific full-length S protein IgG levels were analyzed by validated dipstick with latex immunochromatography assay (dLIA) at Pfizer, Pearl River, NY. SARS-CoV-2 virus neutralization titers were determined by a validated microneutralization assay at Pfizer, Pearl River, NY.

*S-binding IgG assay*

The validated full-length spike (FS) IgG dLIA quantitates IgG antibody levels to the FS protein of SARS-CoV-2. Results were reported in units of immunoglobulin per milliliter (U/mL) calculated from a reference standard with an arbitrarily assigned antibody concentration. The reference standard was present on each assay plate.

The FS protein was coupled to carboxylated Luminex® beads using a two-step carbodiimide reaction. The FS-coupled microspheres were added to a 96-well microtiter assay plate containing appropriately diluted reference standard serum, quality control samples and unknown serum samples. The assay plates were incubated, while shaking, overnight at 2^o^C to 8^o^C. Unbound components were removed by washing and a purified R-Phycoerythrin (PE)-conjugated goat anti-human secondary antibody was added to the reaction wells. The secondary antibody was incubated for 75 to 105 minutes, while shaking, at 18^o^C to 25^o^C. Unbound assay components were removed by washing and the reaction is read on a Luminex® assay plate reader. The PE median fluorescent intensity values measured in each reaction well was directly proportional to the amount of protein-specific IgG in the serum sample.

### SARS-CoV-2 neutralization assay

In brief, heat-inactivated serum samples from participants were serially diluted 1:2 (starting at 1:20) and incubated for 1 hour at 37 °C with live SARS-CoV-2 virus (the virus was derived from the USA_WA1/2020 strain that had been rescued by reverse genetics and engineered to contain a mNeonGreen [mNG] reporter gene in open reading frame 7 of the viral genome that produces green fluorescence upon productive infection of cells) to allow for antigen-specific antibodies to bind to the virus [18]. After 1 hour, the serum/virus mixture was added to Vero cells in 96-well read-out plates (ROPs) and incubated overnight to allow non-neutralized virus to infect the cells. On Day 2, productive viral infection was detected in live cells using the mNeonGreen expressed by the reporter virus. Live cells were stained with the nuclear dye Hoechst 33342. Both viral foci and live cells were enumerated from the ROPs on a Cytation 7 reader (BioTek, USA). A sample titer was calculated as the reciprocal serum dilution at which a specific percentage of the virus is neutralized (eg, 50% of the virus). Samples were run in duplicate and a titer was reported as a GMT of these duplicates.

## Laboratory Procedures: BNT162-01

### Immunogenicity assays

Serum from participants was collected and isolated from whole blood via centrifugation at trial sites, shipped on dry ice, and thawed prior to use in immunogenicity assays. Anti-SARS-CoV-2 S1- and RBD-specific IgG levels were analyzed by ELISA at VisMederi S.r.l., Siena, Italy. SARS-CoV-2 virus neutralization titers were determined by a microneutralization assay based on cytopathic effect (CPE) at VisMederi S.r.l., Siena, Italy. Pseudovirus neutralization assays using VSV-SARS-CoV-2 S pseudovirus were performed at BioNTech SE, Mainz, Germany.

*S1- and RBD-binding IgG assay*

Recombinant SARS-CoV-2 S1 (eEnzyme LLC) or RBD-His protein (Sino Biological) was coated on 96-well ELISA plates. Heat-inactivated serum samples from participants were diluted in 10 two-fold serial dilutions starting at 1:100. Following 1 hour incubation at 37°C, plates were washed with buffer containing 0.05% Tween-20. A secondary horseradish peroxidase (HRP)-conjugated goat anti-human IgG polyclonal antibody (Bethyl Laboratories) was added to the ELISA plate for 30 minutes (min) at 37°C. Plates were washed again, and 3,3’,5,5’tetramethylbenzidine (TMB) substrate was added. After incubation for 20 min at room temperature the reaction was stopped with sulfuric acid. Data were captured as optical density (OD) at 450 nm using an automated microplate reader. The antibody titer was determined by interpolating the cut point using the first dilution that provided an OD read below the predetermined cut-off value of the assay and the first dilution that provided an OD read above the cut-off value of the assay and reported as the GMT of duplicates. Based on the dilutions applied, the lower limit of quantification (LLOQ) was a titer of 100 and the upper limit of quantification (ULOQ) was a titer of 51,200. If no signal above the cut-off value was observed at any dilution, an arbitrary titer value of 50 (half of the LLOQ) was reported. If no signal below the cut-off value was observed at any dilution, an arbitrary titer value of 102,400 (double the ULOQ) was reported. Seroconversion was defined as a minimum 4-fold rise from baseline. If the baseline measurement was below the LLOQ, a post-baseline titer of > 4-fold half of LLOQ was considered seroconversion.

### SARS-CoV-2 neutralization assay

In brief, heat-inactivated serum samples from participants were serially diluted 1:2 (starting at 1:20) and incubated for 1 hour at 37 °C with live SARS-CoV-2 virus (Strain: 2019-nCOV/ITALY-INMI1, identical in sequence to the wild-type SARS-CoV-2 S [Wuhan-Hu-1 isolate]) to allow any antigen-specific antibodies to bind to the virus. Vero E6 cell monolayers were inoculated with the serum/virus mix in 96-well plates and incubated for 3 days to allow infection by non-neutralized virus. The plates were observed under an inverted light microscope and the wells were scored as positive for SARS-CoV-2 infection (i.e., showing cytopathic effect [CPE]) or negative for SARS-CoV-2 infection (i.e., cells were alive without CPE). The neutralization titer was determined as the reciprocal of the highest serum dilution that protected >50% of cells from CPE and reported as GMT of duplicates. If no neutralization was observed, an arbitrary titer value of 5 (half of the limit of detection [LOD]) was reported.

### VSV-SARS-CoV-2 S pseudovirus neutralization assay

A recombinant replication-deficient VSV vector that encodes green fluorescent protein (GFP) and luciferase (Luc) instead of the VSV-glycoprotein (VSV-G) was pseudotyped with SARS-CoV-2 S protein derived from either the wild-type reference strain (NCBI Ref: 43740568) or the VOCs Alpha, Beta, Gamma and Delta according to published pseudotyping protocols. The mutations found in S of the VOCs are listed in **Supplementary Table 7**. In brief, HEK293T/17 monolayers transfected to express SARS-CoV-2 S with the C-terminal cytoplasmic 19 amino acids truncated (SARS-CoV-2-S[CΔ19]) were inoculated with the VSVΔG-GFP/Luc vector. After incubation for 1 hour at 37 °C, the inoculum was removed, and cells were washed with phosphate buffered saline (PBS) before medium supplemented with anti-VSV-G antibody (clone 8G5F11, Kerafast) was added to neutralize residual input virus. VSV-SARS-CoV-2 pseudovirus-containing medium was collected 20 hours after inoculation, 0.2 μm filtered and stored at −80 °C.

For pseudovirus neutralization assays, 40,000 Vero 76 cells were seeded per 96-well. Sera were serially diluted 1:2 in culture medium starting with a 1:15 dilution (dilution range of 1:15 to 1:7,680). VSV-SARS-CoV-2-S pseudoparticles were diluted in culture medium to obtain ~200 transducing units (TU) in the assay. Serum dilutions were mixed 1:1 with pseudovirus for 30 min at room temperature prior to addition to Vero 76 cell monolayers and incubation at 37 °C for 24 hours. Supernatants were removed, and the cells were lysed with luciferase reagent (Promega). Luminescence was recorded, and neutralization titers were calculated as the reciprocal of the highest serum dilution that still resulted in 50% reduction in luminescence. Results were reported as GMT of duplicates. If no neutralization was observed, an arbitrary titer value of 7.5 (half of the LOD) was reported.

### Cell-mediated immunity assays

Whole blood was collected and shipped at ambient temperature to qualified specialty laboratories for peripheral blood mononuclear cell (PBMC) isolation by density gradient centrifugation within 8 hours of blood draw. Cryopreserved PBMCs were shipped in liquid nitrogen and thawed prior to use in cell-mediated immunity (CMI) assays. CMI responses were assayed using *ex vivo* stimulation of PBMCs for flow cytometry/ICS and IFNγ ELISpot using peptides obtained from JPT Peptide Technologies, Berlin, Germany.

### IFNγ ELISpot

IFNγ ELISpot analysis was performed *ex vivo* (without further *in vitro* culturing for expansion) using cryopreserved PBMCs depleted of CD4^+^ and enriched for CD8+ T cells (CD8+ effectors) or depleted of CD8+ and enriched for CD4^+^ T cells (CD4^+^ effectors) using MACS® Technology (Miltenyi). Tests were performed in duplicate and with a positive control (anti-CD3 monoclonal antibody CD3-2 [1:1,000; Mabtech]). Multiscreen filter plates (Merck Millipore) pre-coated with IFNγ-specific antibodies (ELISpotPro kit, Mabtech) were washed with PBS and blocked with X-VIVO® 15 medium (Lonza) containing 2% human serum albumin (CSL-Behring) for 1–5 hours. Per well, 3.3 x 10^5^ effector cells were stimulated for 16–20 hours with overlapping peptide pools representing different portions of the wild-type sequence of SARS-CoV-2 S (N-terminal pools Sp1 [amino acids, aa 1–643] and RBD [aa 1–16 fused to aa 327–528]). Bound IFNγ was visualized using a secondary antibody directly conjugated with alkaline phosphatase followed by incubation with 5-bromo-4-chloro-3′-indolyl phosphate (BCIP)/ nitro blue tetrazolium (NBT) substrate (ELISpotPro kit, Mabtech). Plates were scanned using an AID Classic Robot ELISPOT Reader and analyzed by AID ELISPOT 7.0 software (AID Autoimmun Diagnostika). Spot counts were displayed as mean values of each duplicate. T-cell responses stimulated by peptides were compared to effectors incubated with medium only as a negative control using an in-house ELISpot data analysis (EDA) tool, based on two statistical tests (distribution-free resampling) according to Moodie et al.[32, 33], to provide sensitivity while maintaining control over false positives [15].

To account for varying sample quality reflected in the number of spots in response to anti-CD3 antibody stimulation, a normalization method was applied, enabling direct comparison of spot counts and strength of response between individuals. This dependency was modelled in a log-linear fashion with a Bayesian model including a noise component (unpublished). For a robust normalization, each normalization was sampled 10,000 times from the model and the median taken as normalized spot count value. Likelihood of the model: $l\mathrm{og}\lambda_{E}=\alpha\log\lambda_{P}+\log\beta_{j}+\sigma\varepsilon$ where $\lambda_{E}$ is the normalized spot count of the sample; α is a stable factor (normally distributed) common among all positive controls $\lambda_{P}$; $\beta_{j}$ is a sample *j* specific component (normally distributed); and $\sigma\varepsilon$ is the noise component, of which σ is Cauchy distributed, and ε is Student’s-t distributed. $\beta_{j}$ ensures that each sample is treated as a different batch.

### Flow cytometry/intracellular cytokine staining

Cytokine-producing T cells were identified by ICS. PBMCs thawed and rested for 4 hours in OpTmizer™ medium (Invitrogen) supplemented with 2 µg/mL DNase I (Roche), were restimulated with different portions of the wild-type sequence of SARS-CoV-2 S (N-terminal pools S pool 1 [aa 1-643] and RBD [aa1-16 fused to aa 327-528], and the C-terminal Sp2 [aa 633-1273] (2 µg/mL/peptide; JPT Peptide Technologies) in the presence of GolgiPlug™ (BD) for 18 hours at 37 °C. Controls were treated with dimethyl sulfoxide (DMSO, [AppliChem])-containing medium. Cells were stained for viability and surface markers (CD3 BV421, 1:250; CD4 BV480, 1:50; CD8 BB515, 1:100; all BD Biosciences) in flow buffer (Dulbecco's Phosphate-Buffered Saline [DPBS; Life Technologies] supplemented with 2% fetal bovine serum [FBS; Sigma], 2 mM ethylenediaminetetraacetic acid [EDTA; Sigma-Aldrich] and Brilliant Stain Buffer Plus (BD BioSciences) [according to the manufacturer’s instructions]) or in Brilliant Stain Buffer (BD BioSciences) for 20 min at 4 °C. Afterwards, samples were fixed and permeabilized using the Cytofix/Cytoperm kit according to manufacturer’s instructions (BD Biosciences). Intracellular staining (CD3 BV421, 1:250; CD4 BV480, 1:50; CD8 BB515, 1:100; IFNγ BB700, 1:250; IL-2 PE, 1:10; IL-4 APC, 1:500; all BD Biosciences) was performed in Perm/Wash buffer supplemented with Brilliant Stain Buffer Plus [according to the manufacturer’s instructions] for 30 min at 4°C. Samples were acquired on a fluorescence-activated cell sorter (FACS) VERSE™ or LYRIC™ instruments (BD Biosciences) and analyzed with FlowJo™ software versions 10.6.2, 10.7.1 and 10.8.1 (FlowJo LLC, BD Biosciences). S- and RBD-specific cytokine production was corrected for background by subtraction of values obtained with DMSO-containing medium. Negative values were set to zero. See Supplementary Figure 6 for gating strategy.

*pMHC-class-II multimers*

Using three different pMHC alleles (four for HLA-DRB1*03:01, nine for HLA-DRB1*07:01 and nine for HLA-DRB1*15:01) to detect pMHC- binding. Epitopes show an overlap of all different areas in the S protein- S1, S2 and RBD (Supplementary Figure 8).

### pMHC-class-II multimer staining by flow cytometry

To select MHC-class-II epitopes for multimer analysis a mass-spectrometry-based binding and presentation predictor was used [34, 35] to 8-12 aa-long peptide sequences from S from the GenBank reference sequence for SARS-CoV-2 (accession: NC_045512.2, https://www.ncbi.nlm.nih.gov/nuccore/NC_045512) and paired with 7 MHC-class-II alleles with >5% frequency in the European population. Top predicted epitopes were identified by setting thresholds to the binding percent rank (<1%) and presentation scores (>10^-22^). Peptides were manufactured at 90% purity and (pMHC complexes were tested for refolding and validation as described by Vyasamneni et al. [17]. Combinatorial labelling was used to investigate the antigen specificity of T cells using two-color combinations of five different fluorescent labels to enable detection of up to ten different T-cell populations per sample. For tetramerization, streptavidin (SA)-fluorochrome conjugates were added: SA BV421, SA PE–Cy7 (BD Biosciences); SA BV711, SA APC (Biolegend) and SA PE (ThermoFisher Scientific). For all participants vaccinated with BNT162b2, individualized pMHC class II multimer staining cocktails contained up to ten pMHC complexes, with each pMHC complex encoded by a unique two-color combination. PBMCs (2 × 10^6^) were stained *ex vivo* for 60 min at room temperature with each pMHC multimer cocktail at a final concentration of 4 nM in Brilliant Stain Buffer Plus (BD Horizon). Surface and viability staining was carried out in flow buffer (DPBS (Gibco) with 2% FBS (Sigma), 2 mM EDTA (Sigma-Aldrich)) supplemented with Brilliant Stain Buffer Plus for 30 min at 4 °C. (CD3 BUV395, 1:50; CD45RA BUV563, 1:200; CD8 BV480, 1:200; CD197 BV786, 1:25; CD4 BB515, 1:50 (all BD Biosciences); DUMP channel: CD14 APC–eFluor780, 1:100; CD16 APC–eFluor780, 1:100; CD19 APC–eFluor780, 1:100; fixable viability dye eFluor780, 1:1,667 (all ThermoFisher Scientific)). Cells were fixed for 20 min at 4 °C in 1× stabilization fixative (BD), acquired on a FACSymphony™ A3 flow cytometer (BD Biosciences) and analyzed with FlowJo™ software version 10.8.2 (FlowJo, BD Biosciences). CD4^+^ T-cell reactivities were considered positive when a clustered population was observed that was labelled with only two pMHC multimer colors. See Supplementary Figure 7 for gating strategy.

## Outcomes

The primary outcomes of the BNT162-01 and BNT162-02 trials have been reported elsewhere [6, 15]. BNT162-02 had the exploratory endpoints to describe the safety, tolerability and immunogenicity of BNT162b2 in PWH. All randomized participants who received ≥1 dose of the trial intervention were included in the safety population. All participants in BNT162-02 who used an e-diary to report reactogenicity following vaccination were included in the reactogenicity subset. Safety results of PWH reported here followed the safety reporting algorithm from the wider safety population that was previously reported [6]. Safety data of interest in this cohort was solicited, specific local or systemic AEs within 7 days after the receipt of each dose of vaccine or placebo, as prompted by and recorded in an e-diary in a subset of participants (the reactogenicity subset), and unsolicited AEs (those reported by the participants without prompts from the e-diary) through 1 month after the second dose and unsolicited serious AEs through to unblinding following the second dose.

For the PWH enrolled in the BNT162-01 sub-study, the primary objective was to characterize the long-term adaptive immune response, including functional antibody titers for virus neutralizing titers, fold increase in antibody titers, the number of participants with seroconversion defined as a minimum of 4-fold increase from baseline of neutralizing antibody titers, and CMI responses.

## Statistical Analysis

## *BNT162-02*

The statistical analyses employed in the BNT162-02 trial have been published [6]. Safety endpoints are described as frequencies and proportions. Safety analyses were performed on the safety population which included all randomized participants who received ≥1 dose of vaccine or placebo. Data on local and systemic reactions and any use of medication were collected with e-diaries. Missing reactogenicity e-diary data were not imputed. BNT162-02 employed a Data Monitoring Committee (DMC) responsible for monitoring of participant safety. BNT162-02 is registered with ClinicalTrials.gov (NCT04368728) and complete.

*BNT162-01*

As BNT162-01 does not have a formal statistical hypothesis to test, no formal sample size calculations were performed. The PWH sub-study of BNT162-01 was not powered to a specific endpoint. Safety endpoints are described as frequencies and proportions. Safety analyses provided here are of all enrolled participants who received ≥1 dose of the study intervention. The safety set were defined as all participants who received ≥1 dose of BNT162b2. A subset of HIV-negative participants that completed all visits from the dose escalation part of the trial that received the primary two-dose series vaccination with 30 ug BNT162b2 were included following matching for age and sex as far as feasible. Data are presented for all participants unless otherwise specified. Safety analyses are presented as counts and percentages, and missing data were not imputed. BNT162-01 employed a Safety Review Committee (SRC) rather than a DMC. BNT162-01 is registered with ClinicalTrials.gov (NCT04380701) and is ongoing.

For ICS and MHCII immunogenicity analyses, statistics were calculated using a non-parametric, unpaired Mann-Whitney test. ELISpot analyses were carried out with the EDA tool and Subject Response Evaluation (SURE).

Analyses were performed using SAS (version 9.3 or later) and Prism 9 (GraphPad Software).
